# Community-based screening of Chagas disease among Latin American migrants in a non-endemic country: an observational study

**DOI:** 10.1186/s40249-021-00897-2

**Published:** 2021-09-15

**Authors:** Violeta Ramos-Sesma, Miriam Navarro, Jara Llenas-García, Concepción Gil-Anguita, Diego Torrus-Tendero, Philip Wikman-Jorgensen, María García-López, Concepción Amador-Prous, María-Paz Ventero-Martín, Pedro Guevara-Hernández, Ana Garijo-Saiz, Ares Sanchez-Sanchez, Cristina Bernal-Alcaraz, Ana-Isabel Pujades-Tarraga, Roser Muñoz-Perez, María Flores-Chávez, José-Manuel Ramos-Rincón, the #CorazonesSinChagas Platform, María García-López, Cristina Bernal-Alcaraz, Pedro B. Guevara-Hernández, Jara Llenas-García, Joan Gregori-Colome, Ana Lucas-Dato, Esther Martínez-Birlanga, Estefanía García-Rodríguez, José-Manuel Ramos-Rincón, Diego Torrús-Tendero, M. Paz Ventero-Martín, Adelin Gimeno-Gascón, Ares Sánchez-Sánchez, Roser Muñoz-Pérez, Carmen Almoedo-Albero, Concepción Gil-Anguita, Concepción Amador Prous, Ana-Isabel Pujades-Tárraga, Antonio Santonja, María Sánchez-Valera, Rosa Sánchez-García, Miriam Navarro-Beltrá

**Affiliations:** 1 Internal Medicine Service, HLA Inmaculada Hospital, Granada, Spain; 2 Public Health, Science History and Gynecology Department, Universidad Miguel Hernández de Elche, Alicante, Spain; 3 Epidemiology Unit Public Health Center of Elche, Alicante, Spain; 4 Internal Medicine Department, Hospital Vega Baja de Orihuela (Alicante, Spain)-Foundation for the Promotion of Health and Biomedical Research of the Valencia Region (FISABIO), Alicante, Spain; 5 Clinical Medicine Department, University Miguel Hernández of Elche, Sant Joan d’Alacant, Spain; 6 Internal Medicine Department, Hospital Marina BaixaLa Vila Joiosa (Alicante, Spain)-FISABIO, Alicante, Spain; 7 Internal Medicine Department, General University Hospital of Alicante–Biomedical and Health Research Institute of Alicante (ISABIAL), Alicante, Spain; 8 Parasitology Area, University Miguel Hernández of Elche, Sant Joan d’Alacant, Spain; 9 Internal Medicine Department, University Hospital of Sant Joan, (Sant Joan d’Alacant, Spain)– FISABIO, Alicante, Spain; 10 Microbiology Service, General University Hospital of Alicante–ISABIAL, Alicante, Spain; 11 Pediatric Department, General University Hospital of Alicante–ISABIAL, Alicante, Spain; 12 Digestive Service, General University Hospital of Alicante–ISABIAL, Alicante, Spain; 13 Reference and Research Laboratory in Parasitology, National Center of Microbiology/Mundo Sano Foundation, Madrid, Spain

**Keywords:** Chagas disease, *Trypanosoma cruzi*, Knowledge, Community-based intervention, Migrant, Early diagnosis, Screening

## Abstract

**Background:**

Chagas disease is a parasitic disease endemic to Latin America, but it has become a disease of global concern due to migration flows. Asymptomatic carriers may host the parasite for years, without knowing they are infected. The aim of this study is to assess prevalence of Chagas disease and evaluate the participants’ level of knowledge between Latin American migrants attending a community-based screening campaign.

**Methods:**

Three community-based campaigns were performed in Alicante (Spain) in 2016, 2017 and 2018, including educational chats and blood tests for *Trypanosoma cruzi* serology*.* Participants completed a questionnaire assessing knowledge about the mechanisms of transmission, disease presentation, diagnosis, and treatment. People seropositive for *T. cruzi* underwent diagnostic confirmation by two different tests. Results were analyzed by multivariable logistic regression and expressed as adjusted odds ratios (a*ORs)*, adjusting for age, sex, and time in Spain.

**Results:**

A total of 596 participants were included in the study; 17% were aged under 18 years. Prevalence in adults was 11% [54/496; 95% confidence interval (*CI*): 8.3–14.5%] versus 0% among children. All but one case were in Bolivians. Diagnosis was independently associated with having been born in Bolivia (a*OR*: 102, 95% *CI*: 13–781) and a primary school-level education (a*OR*: 2.40, 95% *CI*: 1.14–5.06). Of 54 people diagnosed with Chagas disease (most of whom were asymptomatic), 42 (77.7%) returned to the clinic at least once, and 24 (44.4%) received treatment. Multivariable analysis showed that coming from Argentina (a*OR*: 13, 95% *CI*: 1.61–1188) or Bolivia (a*OR*: 1.90, 95% *CI*: 1.19–3.39) and having received information about Chagas disease in Spain (a*OR*: 4.63, 95% *CI*: 2.54–8.97) were associated with a good level of knowledge on the disease. Having primary level studies (a*OR*: 0.59, 95% *CI*: 0.34–0.98) and coming from Ecuador (a*OR*: 4.63, 95% *CI*: 2.52–847) were independently associated with a lower level of knowledge.

**Conclusions:**

Community-based interventions are a good strategy for diagnosing neglected diseases such as Chagas disease in non-endemic countries and for identifying and treating infected, asymptomatic individuals.

**Graphic abstract:**

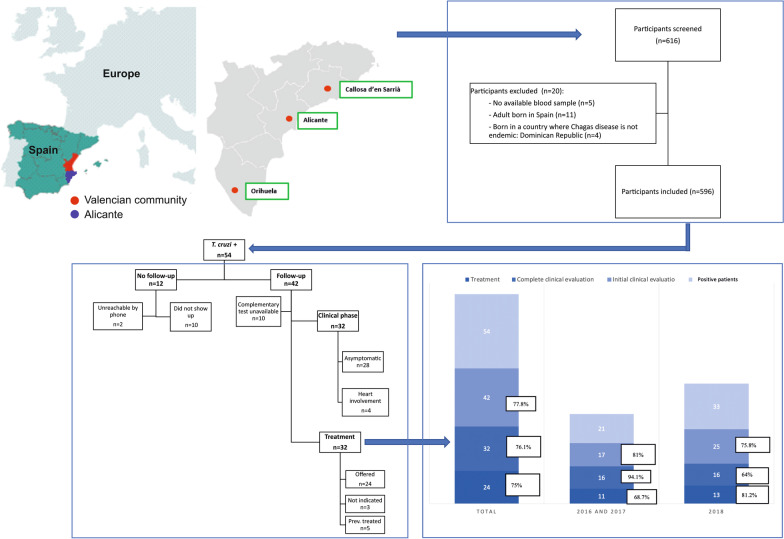

**Supplementary Information:**

The online version contains supplementary material available at 10.1186/s40249-021-00897-2.

## Background

Chagas disease, or American trypanosomiasis, is a systemic chronic parasitic infection caused by the protozoa *Trypanosoma cruzi*, considered a neglected tropical disease [1]. Chagas disease is endemic in 21 continental Latin American countries, where vectorial transmission is the main route of contagion [2].

Vector-borne infections occur only in endemic areas, through the inoculation of *T. cruzi*-infected feces of triatomine bugs into the bite wound. Blood transfusion, organ transplantation, mother-to-infant transmission, and ingestion of contaminated food can be other mechanisms of infection [3]. The classic setting for Chagas disease is rural Latin American areas, such as Bolivia, Colombia, Argentina, or Mexico, where an estimated 6 to 7 million people have Chagas disease and another 70 million are at risk of infection [4].

After an initial parasitemia that can last four to eight weeks, the disease becomes chronic. Up to 70% of infected individuals remain asymptomatic, but they have the parasite and can therefore transmit it through blood transfusions or pregnancy; in non-endemic countries, these are the most frequent transmission routes. Around 30–40% of cases develop clinical manifestations, especially digestive and cardiological ones. Worldwide, complications derived from the disease lead to about 12 000 deaths each year [5].

Approved drugs for treating Chagas disease are benznidazole and nifurtimox, whose efficacy is high during the acute phase (including in congenitally infected newborns) but less so in the chronic phase, when the drugs’ effectiveness in preventing disease progression is unclear [6, 7]. However, recent studies have shown that benznidazole can reduce complications associated with Chagas cardiomyopathy by decreasing the markers of severe cardiomyopathy [8], blood antibodies [9], and mortality [10] and it also is associated with a lower risk of clinical events. Nevertheless, in patients aged older than 55 years, treatment should be individualized to manage the risk of side effects [6, 11].

Migration and specific modes of transmission have made Chagas disease a global issue. Spain is the non-endemic country with the highest prevalence of Chagas disease outside the Americas, with an estimated 65 000 affected individuals, although less than 10% have been diagnosed [12–14]. Migrants from Bolivia have the highest prevalence of the disease in Europe [15].

The absence of symptoms and the negligible perception of disease risk play an important role in the health-seeking behavior of the affected population in non-endemic countries. Different community-based interventions and screening campaigns set up in Spain, other European countries, and the United States of America [16–19] have aimed to reach out to Latin American migrants at risk of Chagas disease, provide them with screening and treatment, and minimize access barriers. Offering culturally tailored information about Chagas disease to affected communities is a key component of these strategies.

In view of the above, a screening campaign for Chagas disease was designed in the framework of a community-based study, performed through a series of workshops. The objective was to assess prevalence of Chagas disease in Latin American migrants attending that event and linkage to care in those with positive screen results. We also evaluate participants’ level of knowledge on Chagas disease and examine the effectiveness of the campaign follow-up interventions.

## Material and methods

### Settings

A community-based screening campaign for Chagas disease and strongyloidiasis was implemented between 2016 and 2018 in Alicante (Spain), a province located on the Mediterranean coast of south-eastern Spain. Around 48 700 migrants from Central and South America are settled in the region [20]**.**

This cross-sectional study had two stages: for the first two years (2016 and 2017), the program was held in the General University Hospital of Alicante, in Alicante city (setting A) and followed up there and in other provincial hospitals. In 2018, the campaign was scaled up into two additional settings, namely, two primary healthcare centers: in Callosa d’en Sarrià, a city located about 40 km north of Alicante city (setting B) and in Álvarez de la Riva in Orihuela, which is 60 km to the south (setting C) (Additional file 1: Figure S1). People with positive serology for *T. cruzi* after screening were followed up in the nearest hospital.

This study is part of a larger research project, entitled "Diagnosis, treatment and follow-up of parasitic diseases (Chagas disease and strongyloidiasis) in the province of Alicante”. The results of the three community-based screening campaigns for strongyloidiasis have recently been reported [21].

### Participants

Inclusion criteria were people from Latin American countries who attended the community-based screening campaigns and signed informed consent. Participants under 18 years of age were included in the Chagas disease screening program if their parents or legal guardian signed the written informed consent, but they were not included in the analysis of disease knowledge. Children born in Spain were included if their parents had been born in Latin America and had travelled to their parents’ country of origin.

Exclusion criteria were: refusal to sign informed consent, no available blood sample, born in a country where Chagas disease is not endemic (e.g., Dominican Republic or Cuba), and adult born in Spain (Additional file 1: Figure S2).

### Questionnaire on Chagas disease knowledge

Once participants signed informed consent, they completed a questionnaire collecting sociodemographic data and epidemiological risk factors for *T. cruzi* infection. The questionnaire also tested participants’ knowledge of Chagas disease through 15 items, based on previously developed questionnaires aimed at populations at risk of Chagas disease and described in a report published by the Spanish Ministry of Health [22]. All documents were in the Spanish language (Additional file 1: File S1).

After completing the questionnaire, participants were invited to listen to a short informative talk about the disease and to resolve doubts with community health workers and healthcare staff. Finally, peripheral blood was drawn to perform *T. cruzi* serology. Medical doctors, medical or nursing students and community health workers were always available for assistance.

To assess the factors associated with the participants’ level of knowledge about Chagas disease, a knowledge index was calculated according to the study published by Romay‑Barja et al. [23]. To create the knowledge index, we selected five facts about Chagas disease that we considered respondents should know: it can be transmitted by triatomines [*vinchuca*s (kissing bugs)] (1 point) or from mother to child (1 point); cardiac involvement is a main consequence of the disease (1 point); digestive disorders constitute the main symptom (1 point); and affected people can be asymptomatic (1 point). Poor knowledge about Chagas disease was defined as the mean score or below. Scores above the mean were considered to indicate a good level of knowledge.

### Serology procedure

Serologic diagnosis of Chagas disease was performed by chemiluminescence immunoassays (CLIA) with *Liaison XL Murex Chagas kit* (DiaSorin, Saluggia, Italy), and an enzyme-linked immunosorbent assay (ELISA)-in-house method using an antigen prepared from a proportional mix of epimastigotes obtained from a culture of three strains of *T. cruzi* (MC, T, Dm 28) in stationary phase. Optical density  > 1.0 titer was considered positive. In case of discordant serology, an indirect in-house immunofluorescent antibody test (IFAT) was performed. The antigen was prepared from cultures of epimastigotes in stationary phase. Titers of 1/80 or more were considered positive. A person was considered infected with *T. cruzi* if the results of the two different serological tests on serum samples were positive. Serology was performed at the Parasitology Department of the National Centre for Microbiology-Instituto de Salud Carlos III (PD-NCM-ISCIII) in Madrid.

### Management of data after the screening campaign and follow-up of people with Chagas disease

Participants with a negative *T. cruzi* serology were informed about the results of the test by ordinary mail to the address provided by them. Positive results were traced, and people were offered an appointment at a specialized outpatient clinic. In 2016 and 2017, outpatient consultations took place in the General University Hospital of Alicante as well in other hospitals, while in 2018 participants were followed up in one of the three participating reference hospitals. Participants’ full medical history was reviewed, and additional tests were ordered to complete the Chagas disease organ involvement study according to each hospital’s protocol. Additional tests could include chest X-ray, electrocardiogram, echocardiography and a molecular diagnostic method [real-time polymerase chain reaction (PCR)—Dia.Pro—Diagnostic Bioprobes Srl, Sesto San Giovanni, Italy], among others.

Specific trypanocide treatment was offered to all Chagas disease patients aged 55 years or less, in the absence of contraindication and based on the current evidence [2, 7, 9, 24]. Response to treatment was defined as PCR results becoming negative in those patients this test was ordered. If PCR was negative at the beginning of the treatment and remain negative once completed, it was considered cure as well. Positive PCRs following treatment were considered failures [25]. The PCR was repeated between three and 18 months after finished the treatment. Medical records of all screened patients in three different hospitals were last reviewed on 7 December, 2019.

### Statistical analysis

Categorical data are presented as absolute numbers and proportions, and continuous variables are expressed as either medians and interquartile ranges (IQRs) or means and standard deviation (*SD*), according to the normality of the distribution. The 95% confidence intervals (*CI*) for prevalence were calculated following the methods described by Newcombe et al. [26]. When appropriate, the chi-square test or Fisher’s exact test were used to compare the distribution of categorical variables, and the Mann–Whitney *U* test or student’s *t* test was used for continuous variables. The measure of association was calculated using the odds ratio (*OR*) with its 95% *CI*. Results were considered statistically significant if the two-tailed *P* value was less than 0.05.

Variables from the crude analysis yielding a *P* value of less than 0.10, plus age, sex, and time in Spain were entered into a multivariable logistic regression using a forward stepwise selection method with the likelihood ratio test. Model validity was evaluated using the Hosmer–Lemeshow test for estimating goodness of fit to the data and its discriminatory ability using the area under the receiver operating curve (AUC). Because there were some missing values, variables that were not recorded for 25% or more of the patients were excluded from the analysis. The results of the regression analysis were expressed as adjusted odds ratios (a*OR*s) with 95% *CI*s. Statistical data analysis was performed using IBM SPSS Statistics for Windows, Version 25 0.0 (IBM Corp; Armonk, NY: USA).

### Ethical aspects

All research was conducted according to the principles expressed in the Declaration of Helsinki, and participation was voluntary. The three community-based screening campaigns (2016–2018) were approved by the Ethics Committee of the General University Hospital of Alicante (Valencia Health Council), Ref: CEIC PI2015 /16 and Ref. CEI PI2018/035, and written informed consent was obtained from all participants. The formal written consent was obtained from the parent/guardian in participants aged under 18 years. Performance and reporting of the study comply with STROBE guidelines (Additional file 1: File S2).

## Results

### Participants

Of 616 people screened, 596 were included in the study: 127 in 2016, 111 in 2017 and 358 in 2018 (setting A, *n* = 124 participants; setting B, *n* = 82; setting C, *n* = 152). One hundred (16.8%) participants were under 18 years of age. Participant characteristics are summarized in Table 1. Most of the non-adult participants were born in Spain (74%), while a plurality of the adults was born in Bolivia (41%).

**Table 1:** Sociodemographic and epidemiological profile of participating adults and children/adolescents

	Adults, *n*/*N* (%)^a^	Children/adolescents, *n*/*N* (%)^a^
Epidemiology and demographic data		
Sex, male	197/496 (39.7)	43/100 (43.0)
Age in years, median (IQR) (*n* = 488)	41 (34–50)	11/100 (9–14)
Years in Spain (IQR) (*n* = 477)	14 (11–16)	NA
Country of birth		
Bolivia	202/496 (40.7)	13 /100 (13.0)
Ecuador	187/496 (37.7)	9 /100 (9.0)
Colombia	65/496 (13.1)	0/100 (0.0)
Argentina	13/496 (2.6)	2 /100 (2.0)
Brazil	7/496 (1.4)	0/100 (0.0)
Paraguay	6/496 (2.6)	0/100 (0.0)
Peru	4/496 (0.8)	0/100 (0.0)
Venezuela	4/496 (0.8)	0/100 (0.0)
Other^b^	8/496 (1.6)	2/100 (2.0)
Spain^c^	-0/496 (0.0)	74/100 (74.0)
Living in rural area	151/201 (75.0)	NA
Highest educational attainment		
Primary school	128/473 (27.1)	NA
Secondary school	265/473 (56.0)	NA
University studies	80/473(16.9)	NA
Healthcare card availability		
Available	360/368 (72.6)	NA
Employment status		
Actively employed	347/464 (70.0)	NA
Unemployed	121/464 (26.1)	NA
Occupation		
Cleaning services	83/308 (16.7)	NA
Services sector^d^	67/308 (13.5)	NA
Construction workers	65/308 (13.1)	NA
Farming	44/308 (8.9)	NA
Hospitality/catering	30/308 (6.0)	NA
Caretakers	19/308 (3.8)	NA
Questions about Chagas disease, *n*/*N* (%) yes		
Have you seen triatomines at home?	108/200 (54.0)	NA
Have you ever had a blood transfusion?	18/199 (14.1)	NA
Do you have relatives with Chagas disease?	38/203 (18.7)	NA
Have you ever heard about Chagas disease?	112/203 (55.2)	NA
Have you been tested before?	58/529 (11.0)	NA

*IQR* interquartile range, *NA* not available

^a^Unless otherwise noted, ^b^Other countries: Mexico (*n* = 2), Honduras (*n* = 2), Nicaragua (*n* = 2), Uruguay (*n* = 2 adults and *n* = 2 children) ^c^Non-adult Spanish participants whose parents were migrants, ^d^Service sector such as: public health and medical services health care, media and communication, financial services, or transportation)

### Prevalence and factors related of Chagas disease

No cases of Chagas disease were detected in participants under 18 years of age (prevalence 0%; 95% *CI*: 0–4.6%), while 54 of the 496 adults were positive for *T. cruzi* infection (prevalence 10.9%, 95% *CI*: 8.3–14.5%; *P* < 0.001).

All but one *T. cruzi* infection were detected in Bolivians. Country of birth in Bolivia and lower educational level were associated with Chagas disease. There were statistically significant differences between participants with or without positive *T. cruzi* serology according to the following variables (*P* < 0.05): age (median 44 vs 41 years), education to primary school (41.5% vs 25.2%), being born in Bolivia (98.1% vs 37.7%), having seen triatomines at home (90% vs 50%), having relatives with Chagas disease (42.9% vs 15.9%), having heard about Chagas disease (95.2% vs 50.5%), previously underwent Chagas disease serology (25.4% vs 8.94%), and having *Strongyloides stercoralis*-positive serology (25.9% vs 10.4%). Being born in Ecuador (0% vs 42.2%), and Colombia (0% vs14.7%) was associated with a significantly lower risk (Table 2).

**Table 2:** Comparison of adult participants with positive versus negative *Trypanosoma cruzi* serology according to sociodemographic and epidemiological variables

	*T. cruzi* positive	*T. cruzi* negative	*P* value
Demographic data			
Sex, male, *n*/*N* (%)	27/54 (50.0)	169/442 (38.2)	0.09
Age (years), median (IQR)	44 (39–51)	41 (34–49)	**0.038**
Time in Spain (years), median (IQR)	12.5 (11–15)	14 (11–16)	0.097
Education, *n*/*N* (%)			
Primary school	22/53 (41.5)	106/420 (25.2)	**0.012**
Secondary school	26,753 (49.1)	239/420 (56.9)	0.28
University studies	5/53 (9.4)	75/420 (17.9)	0.12
Country of birth, *n*/*N* (%)			
Bolivia	53/54 (98.1)	149/442 (37.7)	** < 0.001**
Ecuador	0/54 (0)	187/442 (42.2)	** < 0.001**
Colombia	0/54 (0)	65/442 (14.7)	**0.003**
Argentina	1/54 (1.9)	12/442 (2.7)	0.70
Brazil	0/54 (0)	7/442 (1.6)	0.32
Paraguay	0/54 (0)	6 /442(1.4)	0.38
Peru	0/54 (0)	4/442 (0.9)	0.99
Venezuela	0/54 (0)	4/442 (0.9)	0.99
Otherª	0/54 (0)	8 (1.6)	
Epidemiological data, *n*/*N* (%)			
Living in rural area	16/20 (80.0)	135/181 (74.6)	0.32
Triatomines seen at home	18/20 (90.0)	90/180 (50.0)	**0.001**
Blood transfusion recipient	2/19 (10.5)	26/180 (14.4)	0.64
Relatives with Chagas disease	9/21 (42.9)	29/182 (15.9)	**0.003**
Having heard about Chagas disease	20/21 (95.2)	92/182 (50.5)	** < 0.001**
Positive *Strongyloides stercoralis* serology	14/54 (25.9)	46/442 (10.4)	**0.001**
Previously underwent Chagas disease serology	18/53 (34.0)	40/476 (8.4)	** < 0.001**

*IQR* interquartile range

ªOther countries: Nicaragua (*n* = 2), Uruguay (*n* = 2), Honduras (*n* = 2), Mexico (*n* = 2). In bold, statistically significant differences

After adjusting for age, sex and time in Spain, the multivariable analysis showed that positive *T. cruzi* serology was associated with being born in Bolivia (a*OR*: 102, 95% *CI*: 13–781) and primary school-level education (a*OR*: 2.40, 95% *CI*: 1.14–5.06; Table 3). In this model, the *P* value for the Hosmer–Lemeshow test of goodness of fit was 0.99 with an AUC of 0.91 (95% *CI*: 0.87–0.93).

**Table 3:** Association between sociodemographic and epidemiological characteristics with positive and negative *Trypanosoma cruzi* serology (crude and multivariable analysis)

Variables	Crude *OR* (95% *CI*)	Adjusted *OR* (95% *CI*)	*P* value
Sex, male	1.65 (0.92–2.82)	1.58 (0.77–3.23)	0.21
Age, years	1.02 (1.00–1.53)	1.02 (0.99–1.06)	0.11
Time in Spain, years	0.97 (0.92–1.02)	1.01 (0.93–1.08)	0.89
Primary school	2.10 (1.16–2.78)	2.40 (1.14–5.06)	**0.021**
Country of birth			
*Bolivia*	104 (14.2–761)	102 (13–781)	** < 0.001**
*Ecuador*	NC	NI	
*Colombia*	NC	NI	
Living in rural area	1.36 (0.43–4.28)	NI	
Triatomines seen at home	9.0 (2.03–39.9)	NI	
Blood transfusion recipient	0.69 (0.15–3.19)	NI	
Relatives with Chagas disease	3.95 (1.53–10.2)	NI	
Having heard about Chagas disease	19.5 (2.57–148)	NI	
Positive *Strongyloides stercoralis* serology	3.01 (1.53–5.95)	2.28 (0.96–5.46)	0.062
Previously underwent Chagas disease serology	5.61 (2.91–10.8)	2.12 (0.98–4.50)	0.054

In bold, statistically significant differences; *NC* Not calculable, *OR* Odds ratio. *CI* Confidence interval, *NI* Not included

### Clinical evaluation and management of Chagas disease

**Fig. 1:**
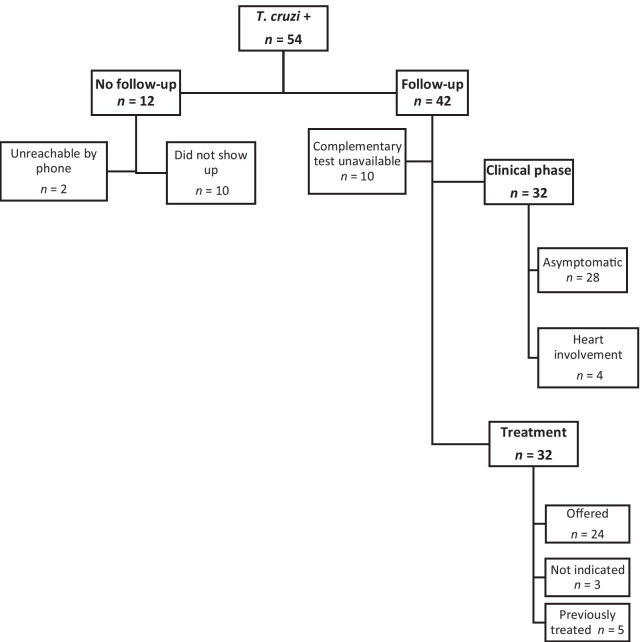
Three years campaigns’ follow chart of the clinical evaluation and management of participants with positive *Trypanosoma cruzi* serology

Thirteen of the 54 participants (24.1%) had been diagnosed with Chagas disease prior to the campaign. Two of the 54 participants with positive *T. cruzi* serology were unreachable by phone, and 10 patients did not show up to the consultation. Thus, medical follow-up was performed in 42 patients (77.8%). Five went to just one appointment, and five more were lost to follow-up after an initial checkup. Thus, no information about the complementary test results was available, so the clinical status of these patients is unknown, and treatment was not offered. Three participants were attended one year after the screening was performed: one from the 2016 campaign and two from 2017. All of them were finally located and examined by a doctor. Twenty-eight of the 32 (87.5%) followed patients were asymptomatic, while 4 of them (12.5%) had Chagasic cardiomyopathy. Of all patients that completed the clinical evaluation (76.2%), treatment was offered to 24 of them (75%). Five (15%) had been treated previously, and three (9%) were not indicated for medication due to age (≥ 55 years) (Fig. 1).

Of the 54 positives, 29 (53.7%) were women and 18 (33.3%) were women of childbearing age (18 to 45 years). Of these, 38.9% (7/18) did not show up to the consultation, while the other 11 were evaluated: 3 (16.7%) were treated before screening and 8 (44.4%) afterward.

The screening campaigns in 2016–2017 and 2018 are described separately, as seropositive participants from 2018 were followed up in three different hospitals, while those from 2016 and 2017 were mostly attended in one. Twenty-one patients were diagnosed with Chagas disease in those two years. Seventeen attended at least one appointment (81%), but one (6%) was lost to follow-up, so 16 completed clinical evaluation (94.1%). Treatment was offered to 11 (68.7%); 4 (25%) had been treated previously (before joining the campaign), and 1 (6.2%) did not meet clinical criteria for parasiticidal treatment.

**Fig. 2:**
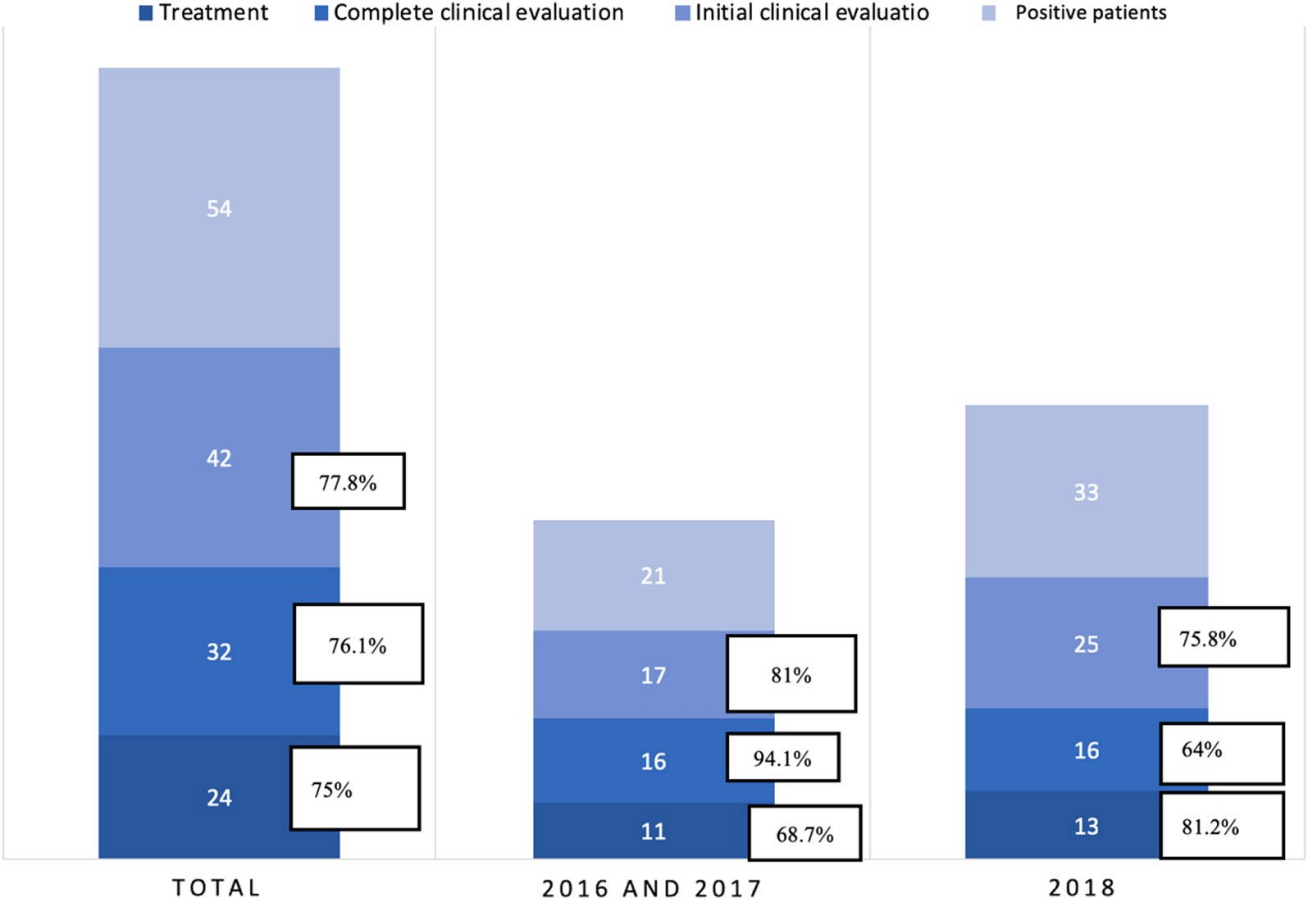
Results of the three campaigns and comparison of two different strategies

In 2018, 33 infected people were diagnosed: 4 cases in setting A, 16 in setting B, and 13 in setting C. Twenty-five (75%) patients attended at least one appointment, but nine (36%) did not complete the clinical evaluation; four patients (20%) were lost to follow-up after the initial evaluation. Altogether, 16 people completed the clinical evaluation (64%). Trypanocide treatment was offered to 13 (81%); 1 (6%) had been treated previously, and 2 (12.5%) were not indicated for treatment (Fig. 2).

### Level of knowledge about Chagas disease

A total of 98.3% (488/496) adult participants included in the study completed the 15-item survey. Not all participants answered the survey questions included in the score. Table 4 shows the survey results.

**Table 4:** Knowledge about Chagas diseases in Bolivians versus non-Bolivians and by *Trypanosoma cruzi* serology

Questions about Chagas disease	Total	Country of birth	Results of *T. cruzi* serology		
		Bolivia	Other Latin American countries	Positive	Negative
Transmission (right answer), *n*/*N* (%)					
Can Chagas disease be transmitted by an insect bite?	310/482 (64.3)	**151/195 (77.4)**	**159/287 (55.4)**^**‡**^	**45/54 (83.9)**	**265/428 (61.9)**^†^
Can Chagas disease be transmitted by kissing bugs?	147/476 (30.9)	**101/193 (52.3)**	**46/283 (16.3)**^**‡**^	**28/54 (51.9)**	**119/422 (28.2)**^†^
Can Chagas disease be transmitted through blood transfusions?	135/448 (30.1)	**72/183 (39.3)**	**63/265 (23.8)**^**‡**^	**23/52 (41.2)**	112/386 (28.3)*
Can Chagas disease be transmitted from mother to child?	179/450 (39.8)	**89 /182 (48.9)**	**90/178 (33.6)**^†^	28/51 (54.9)	151/399 (37.9)
Can Chagas disease be transmitted through organ transplantation?	95/441 (21.5)	43/178 (24.2)	52/263 (19.8)	13/49 (26.5)	82/392 (20.9)
Can Chagas disease be transmitted through sexual contact?	126/446 (28.3)	**54/182 (35.7)**	**81/264 (23.1)**^†^	15/51 (29.4)	111/395 (28.1)
Can Chagas disease be transmitted through by kissing?	159/445 (35.7)	**84/182 (45.2)**	**75/263 (28.5)**^**‡**^	26/51 (51.0)	133/394 (33.8)
Can Chagas disease be transmitted by living with a person who has the disease?	143/446 (32.1)	79/181 (43.6)	64/265 (24.2)	**23/51 (45.1)**	120/395 (30.4)*
Clinical characteristics (right answer), *n*/*N* (%)					
Can Chagas disease affect the heart?	238/488 (48.8)	**129/198 (65.2)**	**109/290 (37.6)**^**‡**^	**39/54 (72.3)**	**199/434 (45.9)**^†^
Can Chagas disease affect the stomach and bowels?	129/471 (27.4)	55/188 (29.39	74/283 (26.1)	14/49 (28.6)	115/422 (27.3)
Can someone with C Chagas disease feel okay (asymptomatic)?	131/473 (27.7)	57/189 (30.2)	74/284 (26.1)	17/50 (34.0)	114/423 (27.0)
Is Chagas disease a serious disease?	289 /482 (60)	**144/192 (75.0)**	**145/290 (50.0)**^**‡**^	37/51 (72.5)	252/162 (58.5)
Diagnosis and treatment (affirmative answer), *n*/*N* (%)					
Is there diagnosis for Chagas disease?	172/399 (43.1)	**89/196 (53.6)**	**83/233 (35.0)**^**‡**^	152/359 (42.2)	20/40 (50)
Is there treatment for Chagas disease?	252/470 (53.6)	**122/189 (64.6)**	**130/281 (46.3)**^**‡**^	**214/419 (51.1)**	**38/51 (74.5)**^†^
Does Chagas disease have a cure?	158/458 (34.5)	65/183 (35.5)	93/275 (33.8)	139/409 (34.0)	19/49 (38.8)

In bold, statistically significant differences: *P-value < 0.05; ^†^*P*-value < 0.01; ^‡^*P*-value < 0.001

More than half (64.3%) of the surveyed participants knew about the vectorial route of transmission, and nearly 40% could correctly respond to the item related to the vertical transmission route. Regarding organ involvement, 48.8% of participants knew that Chagas disease can affect the heart, but only 27.4% were aware of the potential involvement of the digestive tract, and just 27.7% knew that the disease could be asymptomatic (Table 4).

For most items, Bolivians were more knowledgeable than people from other countries; participants with positive *T. cruzi* serology, more so than participants with negative results (Table 4); and people who had attained a higher educational level, more so than those with less schooling (Additional file 1: Table S1). When analyzing the level of the knowledge by gender, women were more aware of the heart involvement (52.5% vs 43.0%; *P* = 0.03), but there were no statistically significant differences regarding knowledge on the vertical transmission route (Additional file 1: Table S1).

The mean knowledge index about Chagas among 488 participants was 1.7 ± 1.7. Argentinians, as a group, were the most knowledgeable (2.8 ± 0.9), followed by people with relatives infected with Chagas disease (2.7 ± 1.6), and those who received information about the disease in Spain (2.7 ± 1.6) (Table 5). Additional file 1: Table S2 shows the variables associated with a good level of knowledge about Chagas disease (score > 2 points) in the bivariable analysis. In the multivariable analysis, only being from Bolivia or Argentina and having received information about Chagas disease in Spain were associated with a good level of knowledge. On the other hand, primary-level studies and being from Ecuador were associated with poorer knowledge (Table 6).

**Table 5:** Mean score (0–5) for survey on general facts about the disease, by sociodemographic indicators

Variables	Median (standard deviation)	*P* value
Sex		0.060
Male	1.5 (1.7)	
Female	1.8 (1.7)	
Age, years		0.421
≤ 41	1.7 (1.7)	
> 41	1.6 (1.6)	
Time in Spain, years		< 0.001
< 14	2.0 (1.6)	
≥ 14	1.3 (1.6)	
Education		< 0.001
Primary school	1.2 (1.5)	
Secondary school	1.8 (1,7)	
University studies	2.2 (1.7)	
Country of birth		< 0.001
Bolivia	2.2 (1.6)	
Ecuador	1.0 (1.5)	
Colombia	1.6 (1.6)	
Argentina	2.8 (0.9)	
Brazil	1.6 (1.5)	
Paraguay	3.0 (1.1)	
Living in rural area		0.521
Yes	1.7 (1.6)	
No	1.6 (1.5)	
Triatomines seen at home		0.002
Yes	2.0 (1.7)	
No	1.3 (1.5)	
Blood transfusion recipient		0.06
Yes	1.1 (1.5)	
No	1.7 (1.7)	
Relatives with Chagas disease		< 0.001
Yes	2.7 (1.6)	
No	1.4 (1.6)	
Having received information about Chagas disease in Spain		< 0.001
Yes	2.7 (1.6)	
No	1.4 (1.6)	
Having heard about Chagas disease		< 0.001
Yes	2.6 (1.5)	
No	0.5 (1.0)

**Table 6:** Association between sociodemographic/epidemiological characteristics and a good level of knowledge about Chagas disease (crude and multivariable analysis)

Variables	Crude *OR* (95% *CI)*	Adjusted *OR* (95% *CI*)	*P* value
Sex, male	0.70 (0.48–1.01)	0.73 (0.47–1.13)	0.17
Age, years	0.98 (0.97–1.00)	0.99 (0.97–1.01)	0.31
Time in Spain, years	0.93 (0.89–0.93)	0.97 (0.93–1.01)	0.18
Primary school	0.43 (0.28–0.66)	0.59 (0.34–0.98)	**0.044**
University studies	1.77 (1.09–2.90)	1.51 (0.85–2.67)	0.11
Country of birth**,** Bolivia	2.79 (1.91–4.04)	1.90 (1.19–3.39)	**0.020**
Country of birth, Ecuador	0.24 (0.16–0.36)	0.51 (0.28–0.94)	**0.031**
Country of birth, Colombia	0.89 (0.52–1.51)	NI	
Country of birth, Argentina	12.58 (1.61–98)	13 (1.61, 1.18)	**0.017**
Living in rural area	1.61 (0.83–3.18)	NI	
Triatomines seen at home	0.48 (0.27–0.85)	NI	
Blood transfusion recipient	0.49 (0.21–1.14)	NI	
Relatives with Chagas disease	4.58 (2.15–10.9)	NI	
Having received information about Chagas disease in Spain	4.38 (2.57–7.44)	4.63 (2.53, 8.47)	**0.023**
Having heard about Chagas disease	14.1 (7.01–29.7)	NI	

In bold, statistically significant differences. *NI* Not included

In the subgroup of Bolivians (*n* = 198), the mean score was 2.2 ± 1.6, with 44.9% showing good knowledge about Chagas disease. In the bivariable analysis, two variables were associated with good knowledge scores: having heard about Chagas (64.9% vs 0.0%; *P* = 0.004) and having received information about the disease in Spain (72.5% vs 37.4%, *P* < 0.001).

## Discussion

Our study showed that one of 10 participants in our screening program have Chagas disease, slightly higher than the pooled prevalence reported in a recent systematic review of Chagas disease in migrants in Europe [18, 27]. All except one came from Bolivia, so one of five Bolivians had Chagas disease. In fact, the factor most strongly associated with Chagas disease was being born in Bolivia. These results are consistent with other community-based seroprevalence studies [18, 27, 28]. People who had stopped studies after primary school were also at higher risk of having Chagas disease, in keeping with other studies of *T. cruzi* antibodies in blood donors in endemic countries [29]. This finding can be linked with the tendency for the disease to affect people with limited resources.

Indeed, Chagas disease is endemic to Latin America and is linked to disadvantaged and poor populations [1]. Pane et al. [27] described housing conditions and building materials as statistically significant factors for acquiring the disease. Close contact with infected animals also favors transmission of the disease [30]. Traditionally associated with rural areas, in recent decades Chagas disease has also been detected in urban settings. Migration from the countryside to the city has favored the growth of urban centers and the appearance of slums [31]. The main control strategies are based on vector control and housing improvements [30]. However, public awareness, an active search for infected persons, and an investment of resources to enable access to the health system and treatment are also key. Each of these barriers presents a unique public health challenge [32]. The persistence of Chagas disease is linked to social, cultural, historical, political and economic processes [30].

One out of four participants with Chagas disease in our study was co-infected with *S. stercoralis*, which is in keeping with the co-infection prevalence of 21% reported in another Spanish Collaborative Network [33, 34].

Of the patients who attended the screening, only 11% had previously undergone serology. A quarter of the participants infected by *T. cruzi* had already tested positive before screening, and 15% had already been treated. Despite this, these participants joined the campaign freely in order to be re-tested. They may not have known about the chronic course of the disease, potentially receiving limited information at the time of the previous test. According to Parsi et al. [35] even after health campaigns in endemic areas, persistent knowledge gaps and misconceptions of serologic test results remain and contribute to creating structural barriers, leading to the normalization and acceptance of Chagas disease and its social consequences. Our campaign suggests that ease of access (testing on weekends, all activities free and freely offered to all) may help to convince people to come to the screening.

Regarding follow-up during the three campaigns, 75% of individuals with positive screening results attended their first medical appointment. As expected, most were in the chronic asymptomatic phase of the disease, and around 75% of them were offered the pharmacological treatment by the time the medical histories were reviewed.

In spite of the advantages of treatment, its administration is inherently complex, since it involves taking several pills twice a day for a recommended time of 60 days. In addition, its use is associated with very frequent adverse effects, which, although mild, sometimes make it necessary to suspend treatment [36]. In endemic areas, these challenges come on top of problems with financing and access to medication [32]. However, with proper coordination between health care services, diagnosis and treatment can be safely implemented even in remote areas. Yun et al. [37] sets out an example of implementing Chagas disease diagnosis and treatment program in resource-limited settings, including remote rural areas, while addressing the limitations associated with drug-related adverse events.

Other studies, like the one by Repetto et al. [38], have reported that less than 30% of participants completed the treatment. Other community-based campaigns that assessed uptake in the screened population show similar data, with a remarkable loss to follow-up: around 50% of diagnosed patients did not begin treatment [39, 40].

Based on the experience in 2016 and 2017, the 2018 campaign was simultaneously organized in three different settings in order to reach more people. This last campaign was successful in terms of engaging a higher number of participants than in the two previous meetings, resulting in a higher number of diagnoses in 2018 compared to the first two years (33 in 2018 vs 21 in 2016–2017). Follow-up of positive-screened individuals was not centralized in a single health care center, as in 2016 and 2017, which could have facilitated access to follow-up and treatment. However, despite the increased access points for follow-up and our best efforts to minimize attrition, a higher number of participants were lost in the final edition.

The phase following campaign implementation was quite challenging, especially in 2018, when more people attended. Participants’ contact details were not always properly collected, and some resided outside Alicante province, among other complications. It seems reasonable that in the 2018 campaign, fewer participants completed the clinical evaluation because of the proximity between the campaign and the data collection.

Globally, migrants face different barriers in attending medical appointments, including precarious jobs, long working hours, and high mobility within and between countries [40–42]. Different strategies have been proposed to avoid loss to follow-up, such administering treatment during the first medical visit, minimizing visits to the hospital, tracing patients with high mobility, and improving communication vertically (primary-hospital care) and horizontally (between hospitals) [17, 43, 44]. Our experience has taught us that the right timing (e.g. avoiding months when migrants typically travel to their countries of origin) is crucial for ensuring participation and reducing time lapses between the event and the first medical visit.

Community screening campaigns are a useful tool for the diagnosis of asymptomatic people. Not only are they a diagnostic strategy, they can also educate the population and raise awareness of the disease as well as bring people closer to the healthcare system and treatment. In addition, these screening programs are typically organized outside working hours to facilitate access to a larger number of people or at events organized by vulnerable communities. Imaz-Iglesia et al. [45] indicates that the active search for infected patients is a cost-effective strategy, especially in women of childbearing age.

These programs reach a significant number of people who undergo serology, and they are often paired with appropriate strategies to get those diagnosed to attend their medical appointments. However, the programs often lack governmental support and are carried out by health professionals working on a voluntary basis. The absence of support from institutions or hospitals can lead to the aforementioned loss of patients during follow-up, since specific consultations are not available, which means an extra effort for the physician.

This is one of the few published quantitative studies focused on the knowledge of Chagas disease in Latin American migrants in Europe and other non-endemic regions. Overall, at-risk populations lack awareness of the disease in non-endemic countries [17, 18]. Ten percent of the participants reached the maximum score of 5 points on the knowledge index, but most scores were below the mean. Among the nearly 200 Bolivian respondents, the mean score (2.2) was above the mean for the whole sample (1.7). This can be attributed to the generalized health education campaigns in Bolivia over the past decades, as this is currently the country with the highest burden of Chagas disease. On the other hand, more than half the Bolivian participants showed poor knowledge on Chagas disease, in line with similar results published recently by Ronay-Barja et al*.* [23] in Madrid and other authors in Spain and Europe, who also report that most people with Chagas disease are from Bolivia [15, 46, 47]. This highlights the need for continuing community-based interventions in order to improve Chagas disease awareness within the population at risk. Fortunately, one factor associated with better knowledge of Chagas disease was “having received information about the disease in Spain.” This highlights the efficacy of the specific information and community-based activities performed across this non-endemic country.

In our setting, where vectorial transmission is not currently possible, few surveyed participants were familiar with that route. These data are consistent with other published studies, which report similar percentages to ours in at-risk populations, both in endemic and non-endemic countries [17, 44, 48]. The vertical transmission route, which is the most relevant in non-endemic areas, has prompted governments to establish protocols for the early detection and treatment of neonatal infections [49–52], although much work remains to be done in this context [28, 51–53].

As mentioned above, the general level of knowledge was low, and few participants responded correctly on the routes of transmission. Women were slightly more aware about vertical transmission than men, although the difference was not statistically significant. This finding suggests an opportunity, as training women from endemic areas could add value to interventions focused on congenital Chagas disease. Indeed, our prior experience has underlined that collaboration with female community health workers can be an important determinant of success of community-based interventions in this area [54].

Less than 30% of participants were aware of the asymptomatic nature of this parasitic infection, one of the main causes of underdiagnosis[16, 35, 40, 55]. Lack of knowledge on Chagas disease in both at-risk populations and healthcare professionals, fear, stigma, and structural barriers are likewise contributing to Chagas disease underdiagnosis [13, 56]. Regarding the educational level, participants who had attained a higher educational level also had a better understanding of the disease.

Strengths of this research include its three-year study period, which enabled us to assess temporal differences in patient profile, diagnosis, and patient follow-up. However, it also has several limitations. First, it used a cross-sectional design, and the setting in a single Spanish province may reduce its generalizability to populations settled in other regions. Most of our participants came from Bolivia, so our sample is less representative of other Latin American countries. However, we consider this to be a strength of the program rather than a limitation: our message and activities are reaching the population that is at the highest risk of Chagas disease in Europe [15]. Second, we encountered some difficulties, discussed above, in following up positive-screened participants. Thirdly, there was no standard Chagas disease protocol in the different healthcare centers where treatment and medical follow-up took place. Lastly, our questionnaire was not validated; it was designed based on a questionnaire used by another Spanish research group [15, 22].

## Conclusions

The community-based intervention implemented in Spain is an effective way of providing access to Chagas disease diagnosis and treatment in vulnerable populations. Work is still needed to develop effective strategies that minimize attrition in order to accompany all patients through the process and to offer them treatment. These activities could contribute to preventing infections in newborns and avoiding organ system complications and suffering in people with chronic infections. Efforts are still needed to raise awareness of Chagas disease, especially in adults from Bolivia and Latin American women of childbearing age.

## Supplementary Information

**Additional file 1: Figure S1.** Map of the region showing the three cities where the community-based screening campaigns were performed. In 2016 and 2017 the program was held in Alicante city (setting **A**) and in 2018, the campaign was scaled up into two primary healthcare centers in Callosa d’en Sarrià, a city located about 40 km north of Alicante city (setting **B**) and Álvarez de la Riva in Orihuela, which is 60 km to the south (setting **C**). **Figure S2. **Participant flow chart. **File S1.** Questionnaire given to participants with sociodemographic data, epidemiological risk factors for *T.cruzi* infection, knowledge and beliefs on CD (Spanish version). **File S2.** STROBE Statement—Checklist of items that should be included in reports of *cross-sectional studies*.** Table S1.** Knowledge about Chagas diseases (CD) by sex and educational level. **Table S2. **Level of knowledge according to sociodemographic factors.

## Data Availability

The datasets analyzed are available in https://github.com/jramosrincon/ChagasScreeninng.

## Abbreviations

a*OR*: Adjusted odds ratio
CLIA: Chemiluminescence immunoassays
ELISA: Enzyme-linked immunosorbent assay
IQR: Interquartile range
IFAT: Immunofluorescent antibody test
*OR*: Odds ratio
PD-NCM-ISCIII: National Centre for Microbiology-Instituto de Salud Carlos III (PD-NCM-ISCIII)
NC: Not calculable
NI: Not included
*SD*: Standard deviation
