# Supplementary material for: Community-based screening of Chagas disease among Latin American migrants in a non-endemic country: an observational study
Source: Infect Dis Poverty. 2021 Sep 15;10:117. doi: 10.1186/s40249-021-00897-2 (PMC8441044; doi:10.1186/s40249-021-00897-2)
Supplement: Supplementary file 1 — Additional file 1: Figure S1. Map of the region showing the three cities where the community-based screening campaigns were performed. In 2016 and 2017 the program was held in Alicante city (setting A) and in 2018, the campaign was scaled up into two primary healthcare centers in Callosa d’en Sarrià, a city located about 40 km north of Alicante city (setting B) and Álvarez de la Riva in Orihuela, which is 60 km to the south (setting C). Figure S2. Participant flow chart. File S1. Questionnaire given to participants with sociodemographic data, epidemiological risk factors for T.cruzi infection, knowledge and beliefs on CD (Spanish version). File S2. STROBE Statement—Checklist of items that should be included in reports of cross-sectional studies. Table S1. Knowledge about Chagas diseases (CD) by sex and educational level. Table S2. Level of knowledge according to sociodemographic factors. [file 40249_2021_897_MOESM1_ESM.docx]

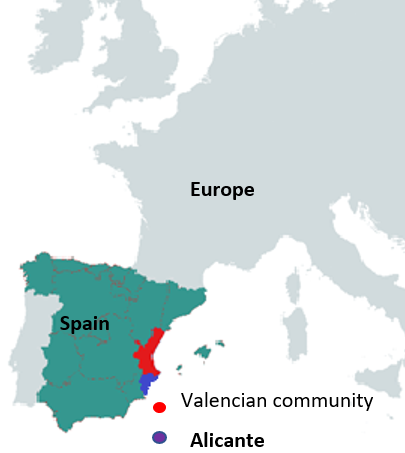

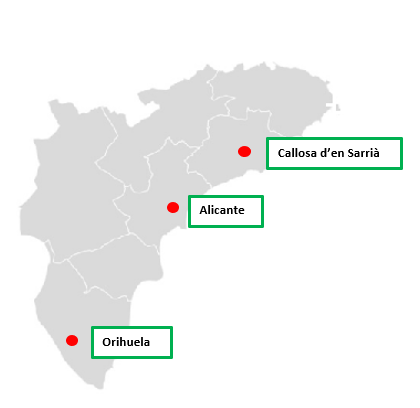


**Figure S1.** Map of the region showing the three cities where the community-based screening campaigns were performed. In 2016 and 2017 the program was held in Alicante city (setting A) and in 2018, the campaign was scaled up into two primary healthcare centers in Callosa d’en Sarrià, a city located about 40 km north of Alicante city (setting B) and Álvarez de la Riva in Orihuela, which is 60 km to the south (setting C)

**Figure S2.** Participant flow chart

**File S1:** questionnaire given to participants with sociodemographic data, epidemiological risk factors for *T.cruzi* infection, knowledge and beliefs on CD (Spanish version).

**Cuestionario sobre las características epidemiológicas y el grado de conocimiento sobre enfermedades parasitarias entregado a la población inmigrante:**

Nombre y Apellidos: _______________________________________________________

Fecha de nacimiento: __/__/__ Teléfono: ___________________

___________________

Sexo: Varón  Mujer  Tarjeta sanitaria: Sí  No 

País de origen: _____________ Zona: ________________

Fecha de llegada a España (día/mes/año): __/__/__

Nivel de estudios: Nunca fui a escuela  Primaria  Secundaria  Universidad 

¿Actualmente tienes trabajo? Sí No ¿Cuál?_________________

¿Tienes hijos? Sí No  ¿Cuántos?: _________

¿Has vivido o pasado temporadas en zona de campo en tu país? Sí No 

¿Recuerdas haber visto *vinchucas* o “chinches besucones” allí? Sí No  No sé

¿Has recibido transfusiones de sangre? Sí No  No sé 

¿Algún familiar afectado por la enfermedad de Chagas? Sí No  No sé 

- ¿Cuáles?: ­­­­­­­­­­­­­­­­­­­­­______________________

¿Te has hecho alguna vez la prueba del Chagas?: Sí No  No sé 

- ¿Resultado?: Tengo la enfermedad de Chagas  No tengo la enfermedad 

**¿Cómo supiste de esta actividad?**

 Me dieron un cartel en mano. Lugar: ____________________________

 Vi un cartel colgado. Lugar: ____________________________

Cancha Locutorio Restaurante Tienda Peluquería

Centro de salud Parada de bus/Tram Otros: __________

 Periódico. ¿Cuál?: ______________________

 Radio. ¿Cuál?: ______________________

 Redes sociales (Facebook, Twitter, web…)

 Otros: ­­­­­­­­­­­­­­­­­­­­­­______________________________

**1. ¿Has oído hablar de la estrongiolidiasis o del estrongiloides?**  Sí  No 

¿Dónde? ________________________________________________________________

**2. ¿Has oído hablar de la enfermedad de Chagas?** Sí  No 

¿Dónde? ________________________________________________________________

**3 ¿Has recibido información sobre esta enfermedad en España?** Sí  No 

¿Dónde? ________________________________________________________________

**4. ¿Sabes cómo se transmite la enfermedad de Chagas?**

- Picadura de un insecto Sí No  No sé 

¿Qué insecto?:______________________________

- Por transfusión de sangre ……………………… Sí No  No sé 

- De una mujer embarazada a su bebé …… Sí No  No sé 

- Por trasplante de un órgano ………………… Sí No  No sé 

- Por relaciones sexuales …………………………… Sí No  No sé 

- Al besar a una persona …………………………… Sí No  No sé 

- Por convivir con una persona con Chagas … Sí No  No sé 

- Otras: ________________________________________

**5. ¿Sabes qué daños puede producir en el cuerpo la enfermedad de Chagas?**

- Afecta al corazón ……………………………… Sí No  No sé 

- Afecta al estómago e intestinos …………… Sí No  No sé 

- Puedes tener la enfermedad y no tener ningún síntoma Sí No  No sé 

- Otros: _____________________________________________

**6. ¿Crees que es una enfermedad grave?** ………………… Sí No  No sé 

**7. ¿Se puede diagnosticar?** …………………………………… Sí No  No sé 

- Cómo? ___________________________________________

**8. ¿Tiene tratamiento?** ……………………………………… Sí No  No sé 

- ¿Sabes qué tipo de tratamiento y cómo se llama? _________________________

- ¿Crees que se cura?: …………………………… Sí No  No sé 

**9. ¿Qué significa para ti la enfermedad de Chagas?**

___________________________________________________________________________________________________________________________________________________

**File 2S. STROBE Statement—Checklist of items that should be included in reports of *cross-sectional studies***

|  | Item  No | Recommendation | Pag No |
| --- | --- | --- | --- |
| **Title and abstract** | 1 | (*a*) Indicate the study’s design with a commonly used term in the title or the abstract | 1 |
|  |  | (*b*) Provide in the abstract an informative and balanced summary of what was done and what was found | 3 |
| Introduction | | |  |
| Background/rationale | 2 | Explain the scientific background and rationale for the investigation being reported | 5 |
| Objectives | 3 | State specific objectives, including any prespecified hypotheses | 6 |
| Methods | | |  |
| Study design | 4 | Present key elements of study design early in the paper | 6 |
| Setting | 5 | Describe the setting, locations, and relevant dates, including periods of recruitment, exposure, follow-up, and data collection | 6 |
| Participants | 6 | (*a*) Give the eligibility criteria, and the sources and methods of selection of participants | 7 |
| Variables | 7 | Clearly define all outcomes, exposures, predictors, potential confounders, and effect modifiers. Give diagnostic criteria, if applicable | 7-8 |
| Data sources/ measurement | 8* | For each variable of interest, give sources of data and details of methods of assessment (measurement). Describe comparability of assessment methods if there is more than one group | *8* |
| Bias | 9 | Describe any efforts to address potential sources of bias | 8 |
| Study size | 10 | Explain how the study size was arrived at | 8 |
| Quantitative variables | 11 | Explain how quantitative variables were handled in the analyses. If applicable, describe which groupings were chosen and why | 9 |
| Statistical methods | 12 | (*a*) Describe all statistical methods, including those used to control for confounding | 9 |
|  |  | (*b*) Describe any methods used to examine subgroups and interactions |  |
|  |  | (*c*) Explain how missing data were addressed |  |
|  |  | (*d*) If applicable, describe analytical methods taking account of sampling strategy |  |
|  |  | (*e*) Describe any sensitivity analyses |  |
| Results | | |  |
| Participants | 13* | (a) Report numbers of individuals at each stage of study—eg numbers potentially eligible, examined for eligibility, confirmed eligible, included in the study, completing follow-up, and analysed | 10 |
|  |  | (b) Give reasons for non-participation at each stage | 11 |
|  |  | (c) Consider use of a flow diagram |  |
| Descriptive data | 14* | (a) Give characteristics of study participants (eg demographic, clinical, social) and information on exposures and potential confounders | 11-12 |
|  |  | (b) Indicate number of participants with missing data for each variable of interest |  |
| Outcome data | 15* | Report numbers of outcome events or summary measures | 13 |
| Main results | 16 | (*a*) Give unadjusted estimates and, if applicable, confounder-adjusted estimates and their precision (eg, 95% confidence interval). Make clear which confounders were adjusted for and why they were included | 13 |
|  |  | (*b*) Report category boundaries when continuous variables were categorized | 14 |
|  |  | (*c*) If relevant, consider translating estimates of relative risk into absolute risk for a meaningful time period |  |
| Other analyses | 17 | Report other analyses done—eg analyses of subgroups and interactions, and sensitivity analyses | 17 |
| Discussion | | |  |
| Key results | 18 | Summarise key results with reference to study objectives | 18 |
| Limitations | 19 | Discuss limitations of the study, taking into account sources of potential bias or imprecision. Discuss both direction and magnitude of any potential bias | 21 |
| Interpretation | 20 | Give a cautious overall interpretation of results considering objectives, limitations, multiplicity of analyses, results from similar studies, and other relevant evidence | 21 |
| Generalisability | 21 | Discuss the generalisability (external validity) of the study results | 21 |
| Other information | | |  |
| Funding | 22 | Give the source of funding and the role of the funders for the present study and, if applicable, for the original study on which the present article is based | 22 |

**Note:** An Explanation and Elaboration article discusses each checklist item and gives methodological background and published examples of transparent reporting. The STROBE checklist is best used in conjunction with this article (freely available on the Web sites of PLoS Medicine at http://www.plosmedicine.org/, Annals of Internal Medicine at http://www.annals.org/, and Epidemiology at http://www.epidem.com/). Information on the STROBE Initiative is available at www.strobe-statement.org.

**Table S1.** Knowledge about Chagas diseases (CD) by sex and educational level

| **Questions about CD** | **Total**  **n/N (%)** | **Sex** | | **Educational level** | | |
| --- | --- | --- | --- | --- | --- | --- |
|  |  | **Female**  **n/N (%)** | **Male**  **n/N (%)** | **Primary school**  **n/N (%)** | **High school**  **n/N (%)** | **University studies**  **n/N (%)** |

| **Transmission (right answer)** |
| --- |

| Can CD be transmitted by an insect bite? | 310/482 (64.3) | 196/294 (66.7) | 114/188 (60.8) | 67/126 (21.7) | 179/263 (57.9) | **63/83 (20.4)***** |
| --- | --- | --- | --- | --- | --- | --- |
| Can CD be transmitted by kissing bugs? | 147/476 (30.9) | 98/293 (33.4) | 49/183 (26.8) | 29/126 (19.7) | 82/259 (55.8) | 36/81 (24.5)** |
| Can CD be transmitted through blood transfusions? | 135/448(30.1) | 85/271 (31.4) | 50/177 (28.2) | 27/124 (19.6) | 84/248 (60.9) | 27/70 (19.6)* |
| Can CD be transmitted from mother to child? | 179/450 (39.8) | 112/276 (40.6) | 67/107 (38.5) | 36/126 (20.1) | 105/246 (58.7) | 38/74 (21.2)** |
| Can CD be transmitted through organ transplantation? | 95/441 (21.5) | 63/270 (23.3) | 32/171 (18.7) | 23/123 (23.5) | 58/243 (59.2) | 17/69 (17.3) |
| Can CD be transmitted through sexual contact? | 126/446 (28.3) | 74/269 (27.5) | 52/177 (29.4) | 23/125 (18) | 75/246 (58.6) | **30/69 (23.4)***** |
| Can CD be transmitted through by kissing? | 159/445 (35.7) | 97/268 (36.2) | 62/177 (35) | 29/124 (18) | 97/246 (60.2) | **35/69 (21.7)***** |
| Can CD be transmitted by living with a person who has the disease? | 143/446 (32.1) | 90/269 (33.5) | 53 /177 (29.9 | 29/124 (20.1) | 82/246 (56.9) | 33/69 (22.9)** |

| **Clinical characteristics (right answer)** |
| --- |

| Can CD affect the heart? | 238/488 (48.8) | **155/295 (52.5)** | **83/193 (43.0)*** | 45/129 (19.1) | 135/266 (57.4) | **55/84 (23.4)***** |
| --- | --- | --- | --- | --- | --- | --- |
| Can CD affect the stomach and bowels? | 129/471 (27.4) | 84 / 285 (29.5) | 45/186 (24.2) | 23/127 (18) | 75/255 (58.6) | 30/81 (23.4)** |
| Can someone with CD feel okay (asymptomatic)? | 131/473 (27.7) | 83/289 (28.7) | 48/184 (46.1) | 22/127 (16.7) | 80/257 (60.6) | 30/80 (22.7)** |
| Is CD a serious disease? | 289 /482 (60) | 185/292 (63.4) | 104/190 (54.7) | 60/126 (21.1) | 163/265 (57.4) | 61/82 (21.5) ** |

| **Diagnosis and treatment (answer yes)** |
| --- |

| Is there diagnosis for CD? | 172/399 (43.1) | 111/245 (45.3) | 61/154 (39.4) | 61/128 (20.3) | 176/267 (58.7) | **63/80 (21)***** |
| --- | --- | --- | --- | --- | --- | --- |
| Is there treatment for CD? | 252/470/ (53.6) | 161/285 (56.5) | 91/185 (49.2) | 55/125 (22.1) | 148/259 (59.4) | 46/77 (18.5)* |
| Does CD have a cure? say yes | 158/458 (34.5) | 100/279 (35.8) | 58/179 (32.4) | 35/121 (22.3) | 93/251 (59.2) | 29/77 (18.5) |

In bold, statistically significant differences: * P-value <0.05; ** P-value <0.01; *** P-value <0.001

**Table S2.** Level of knowledge according to sociodemographic factors

| **Variables** | **Low knowledge of Chagas disease** | **Good knowledge of Chagas disease** | **P value** |
| --- | --- | --- | --- |
| **Demographic data** |  |  |  |
| Sex, male, n (%) | 111/193 (57.5) | 82/193 (42.5) | 0.060 |
| Age in years, median (IQR) | 43 (35-50) | 41 (34-48) | 0.097 |
| Years in Spain, median (IQR) | 15 (12-17) | 12 (9-15) | **<0.001** |
| **Education, n/N (%)** |  |  |  |
| Primary school | 85/128 (66.4) | 43/128 (33.6) | **<0.001** |
| Secondary school | 126/261 (48.3) | 135/261 (51.7) | 0.84 |
| University studies | 32/80 (40) | 48/80 (60.0) | **0.020** |
| **Country of birth, n/N (%)** |  |  |  |
| Bolivia | 74/198 (37.4) | 124/198 (62.6) | **<0.001** |
| Ecuador | 135/185 (73.0) | 50/185 (27.0 | **<0.001** |
| Colombia | 35/64 (54.7) | 29/64 (45.3) | 0.67 |
| Argentina | 1/12 (8,3) | 11/12 (91.7) | **0.02** |
| Brazil | 4/7 (57.1) | 3/7/ (42.9) | 0.79 |
| Paraguay | 1/6 (16.7) | 5/6 (83.3) | 0.079 |
| **Epidemiological data, n/N (%)** |  |  |  |
| Living in rural area | 75/149 (50.3) | 74/149 (49.7) | 0.52 |
| Triatomines seen at home | 48/107 (44.9) | 59/107 (55.1) | **0.012** |
| Blood transfusion recipient | 19/28 (67.9) | 9/28 (32.1) | 0.096 |
| Relatives with Chagas disease | 9/38 (23.7) | 29/38 (76.3) | **<0.001** |
| Received info. about Chagas disease in Spain | 21/87 (24.1) | 66/87 (75.9) | **<0.001** |
| Heard about Chagas disease | 31/111 (27.2) | 80/111 (72.1) | **<0.001** |
